# Austrian recommendations for best clinical practice in case of haemorrhagic traumatic brain injury under platelet inhibitors or non-vitamin K antagonist oral anticoagulants: an additional therapeutic option to consider

**DOI:** 10.1186/s13054-020-02922-6

**Published:** 2020-05-08

**Authors:** Patrick M. Honore, Aude Mugisha, Luc Kugener, Sebastien Redant, Rachid Attou, Andrea Gallerani, David De Bels

**Affiliations:** ICU Department, Centre Hospitalier Universitaire Brugmann-Brugmann University Hospital, Place Van Gehuchtenplein, 4, 1020 Brussels, Belgium

We read with great interest the recent paper by Wiegele et al. who describe treatment options in the case of traumatic brain injury (TBI), especially haemorrhagic TBI, in patients receiving oral anticoagulant therapy [1]. We would like to expand somewhat on the therapeutic options they described, in particular regarding ticagrelor and new oral anticoagulants (NOACs). For ticagrelor, a new therapeutic option may be the use of the CytoSorb device. Indeed, CytoSorb can efficiently remove anti-platelet agents in order to restore normal platelet function and to stop bleeding wherever it is occurring [2]. In their study, Angheloiu et al. were able to remove 99% of ticagrelor from human blood in less than 4 h when using CytoSorb [2]. The specific monoclonal antibody reversal agent for ticagrelor is not yet available at the bedside [3]. In the future, both therapies could be used to complement each other. For reversal of NOACs, CytoSorb may represent an effective, accessible and easy to use alternative to antidotes, which are often very expensive and not always available. In an experimental work by Koertge et al., it was found that more than 91% of rivaroxaban could be removed from the blood during 1 h use of CytoSorb [4]. This new therapy could perhaps complement the use of the antidote andexanet alfa, particularly if the antidote is not immediately available. In conclusion, we believe that studies comparing the two strategies (sorbents versus monoclonal antibodies) are urgently needed and that the use of CytoSorb to remove NOACs and anti-platelet agents in order to restore normal coagulation and to stop bleeding would be a very useful addition to the information presented in the Austrian recommendations.

## Authors’ response

Herbert Schöchl, Marion Wiegele, Eva Schaden

To the editor

We would like to thank Honore et al. for their interest in the Austrian interdisciplinary consensus statement on diagnosis and treatment of traumatic brain injury (TBI) patients on oral anticoagulants. The author state that bleeding patients on ticagrelor and non-vitamin K antagonist oral anticoagulants (NOACs) might benefit from extracorporeal removal of these drugs using CytoSorb® haemoperfusion (CHP). Indeed, in emergency open-heart surgery CHP of ticagrelor and rivaroxaban resulted in reduced bleeding complications and less drainage volume compared to a historical control group [5].

Neither platelet transfusion nor desmopressin has been proven to be efficient in ticagrelor-associated bleeding. An in vitro study revealed that CHP removed > 99% of ticagrelor from human blood samples within 3 h [2]. Albumin represents an alternative approach to bind ticagrelor. An experimental study using high-dose albumin spiking of blood samples containing ticagrelor resulted in a significant improvement of platelet function [6]. This might be considered a less invasive and more rapid option compared to CHP.

The role of CHP as an effective and easy to use alternative for NOAC removal in major bleeding is currently unproven. Experimental data revealed that within 1 h of CHP, 91.6% of rivaroxaban was effectively eliminated from the blood [4]. No data for edoxaban and apixaban or for the thrombin inhibitor dabigatran have been published so far.

For dabigatran reversal, the humanised antibody fragment idarucizumab has been proven efficient. The drug is widely available and its cost is acceptable. Thus, idarucizumab clearly represents the therapy of choice in dabigatran-related bleeding. The evidence is less clear for the specific Xa inhibitor antagonist andexanet alfa. The drug costs are considerable, prothrombotic side effects have been reported and the clinical efficacy of andexanet alfa is not fully proven. A current meta-analysis revealed that prothrombin complex concentrate (PCC) showed comparable haemostatic efficacy to andexanet alfa, but PCC is currently not approved for Xa-inhibitor reversal [7]. Thus, before suggesting CHP in bleeding TBI patients, we would highly recommend PCC as a more rapid, widely available, and less invasive alternative for Xa-inhibitor reversal compared to CHP.

We agree with Honore et al. that CHP might represent an interesting alternative to eliminate ticagrelor. For bleeding patients under NOACs, a variety of specific and unspecific reversal agents are available. Thus, before recommending an invasive procedure such as CHP in TBI patients, both safety and efficacy have to be confirmed in vivo.

## Data Availability

Not applicable.

## Abbreviations

TBI: Traumatic brain injury
DDAVP: Desmopressin
NOACs: New oral anticoagulants
